# The Utstein template for uniform reporting of data following major trauma: A joint revision by SCANTEM, TARN, DGU-TR and RITG

**DOI:** 10.1186/1757-7241-16-7

**Published:** 2008-08-28

**Authors:** Kjetil G Ringdal, Timothy J Coats, Rolf Lefering, Stefano Di Bartolomeo, Petter Andreas Steen, Olav Røise, Lauri Handolin, Hans Morten Lossius, Utstein TCD expert panel

**Affiliations:** 1 Department of Research, Norwegian Air Ambulance Foundation, Drøbak, Norway; 2 Faculty of Medicine, Faculty Division Ullevål University Hospital, University of Oslo, Norway; 3 Academic Unit of Emergency Medicine, Leicester University, UK; 4 Institute for Research in Operative Medicine, University of Witten/Herdecke, Cologne-Merheim Medical Centre, Cologne, Germany; 5 Unit of Hygiene and Epidemiology, DPMSC, School of Medicine, University of Udine, Italy; 6 Orthopaedic Centre, Ullevål University Hospital, Oslo, Norway; 7 Department of Orthopaedics and Traumatology, Helsinki University Central Hospital, Finland

## Abstract

**Background:**

In 1999, an Utstein Template for Uniform Reporting of Data following Major Trauma was published. Few papers have since been published based on that template, reflecting a lack of international consensus on its feasibility and use. The aim of the present revision was to further develop the Utstein Template, particularly with a major reduction in the number of core data variables and the addition of more precise definitions of data variables. In addition, we wanted to define a set of inclusion and exclusion criteria that will facilitate uniform comparison of trauma cases.

**Methods:**

Over a ten-month period, selected experts from major European trauma registries and organisations carried out an Utstein consensus process based on a modified nominal group technique.

**Results:**

The expert panel concluded that a New Injury Severity Score > 15 should be used as a single inclusion criterion, and five exclusion criteria were also selected. Thirty-five precisely defined core data variables were agreed upon, with further division into core data for Predictive models, System Characteristic Descriptors and for Process Mapping.

**Conclusion:**

Through a structured consensus process, the Utstein Template for Uniform Reporting of Data following Major Trauma has been revised. This revision will enhance national and international comparisons of trauma systems, and will form the basis for improved prediction models in trauma care.

## Background

### The Utstein template for uniform reporting of data following major trauma

To permit data collection and statistics on major trauma care, in 1999 a working group from the International Trauma Anaesthesia and Critical Care Society (ITACCS) published a recommendation for the Utstein Template for Uniform Reporting of Data following Major Trauma [1]. The template extracted data for the pre-hospital phase, early in-hospital management, and for co-morbidity and outcome. In accordance with the previous Utstein templates, it was commended that data were to be classified as 'Core' (essential) or 'Optional' (supplemental). Despite the intention of facilitating studies to improve the understanding of trauma and trauma care, only a few papers have been published based on the template [2,3]. This indicates a need for further development, and in particular, a major reduction in the large number (92) of core data variables [1], as well as the addition of more precise definitions of these variables [3].

### Trauma registries

Due to the practical difficulties with performing randomised controlled trials in severe trauma cases, valid scientific evidence is often lacking. Systematic prospective registry-based data collection for documenting trauma care is performed by several local, regional and national trauma registries. However, such registries cannot replace randomised clinical trials, but allow for exploration of relationships present in the collected data. The primary aims of these trauma registries are to enable comparative analyses of trauma care and outcome to provide quality improvement and optimal care of the injured patients [4]. The development of a European trauma registry may provide population-based comprehensive data on trauma incidence, epidemiology and trends. Further, it may enable development of regional outcome prediction models (taking special European factors into consideration) and thus set baseline norms for future trauma outcome studies. In Europe, there has been some reluctance to share local and national data, but it is recognised that lessons learned in one area of Europe may be useful for other European states [5]. However, when comparison is conducted, it is important to ensure that the reasons for differences in outcome are due to differences in the quality of trauma care or to differences in trauma systems, and not to variations in population characteristics [6].

### TRISS methodology

Over the last two decades, the Trauma and Injury Severity Score (TRISS) method [7,8], with coefficients for prediction of outcome has been the most commonly used method for comparison of outcome in trauma patients. The TRISS coefficients were originally derived from the United States Major Trauma Outcome Study (US MTOS) [9,10] but more recently the coefficients have been updated based on patient cases from the National Trauma Data Bank [11]. However, the TRISS method has some limitations, and it has been criticised by many authors [7,12-21]. Among other things, the TRISS model requires scoring the Revised Trauma Score (RTS) [22] components (Glasgow Coma Scale [GCS] [23], respiratory rate [RR] and systolic blood pressure [SBP]) on admission in the emergency department (ED), and does not take into account co-morbidity. Despite its limitations, TRISS continues to be the most accepted and widely-used tool for comparing trauma outcome in North America and in some parts of Europe.

### Comparing and benchmarking European trauma care

In Europe, the UK Trauma Audit and Research Network (TARN) [24], along with the Trauma Registry of the German Society of Trauma Surgery (DGU-TR) [25], represent the largest trauma registries. There has also been a move towards developing a European Trauma Audit and Research Network (EuroTARN) [26], and a core dataset with inclusion and exclusion criteria has been created. Nevertheless, to date, no consensus has been reached between countries on the details and extent of the dataset. A first report from EuroTARN concluded that it is possible to collect data from established trauma registries, and the initial analysis revealed significant international variation [5]. As a continuation of this effort, a European project has been initiated by the DGU-TR, UK TARN and the Scandinavian Networking Group for Trauma and Emergency Management (SCANTEM) [27], for developing a joint European Core Dataset (EuroCoreD) for a future European Trauma Registry.

### The 2007 revision of the Utstein template for uniform reporting of data following major trauma

Despite significant efforts [1,5], comparison of trauma care and outcome within Europe has not yet been carried out in a systematic way, mainly because inclusion criteria, data definitions and coding formats vary significantly between registries, and also because patient selection is not comparable [5,28]. Further efforts to establish uniform and standardised inclusion and exclusion criteria, as well as a minimum list of core data variables with precise definitions, are essential [3,5]. In addition, consistent methods of injury scoring need to be agreed upon [4,29-31]. To address this need for a European consensus, SCANTEM, TARN, DGU-TR and the Italian National Registry of Major Injuries (RITG) [32] carried out a consensus process, concluding with symposia in May and December 2007 at the Utstein Abbey [33], Norway. Selected experts met with the aim of further developing the Utstein Template for Uniform Reporting of Data following Major Trauma. At that time, they defined inclusion and exclusion criteria, and a minimum core dataset with precise definitions. In addition, the aim of the revised template was to develop a standard for comparison of trauma data that was compatible with the large trauma registries in Europe, also adhering to EuroTARN. The template was intended to support the establishment of a European Trauma Registry, promote further development of a European model for outcome prediction and allow European and international trauma auditing and benchmarking.

## Methods

This revision of the Utstein template is based on a nominal group technique (NGT) process [34,35] modified to fit the purpose. For participation in the NGT process, a European expert panel was selected.

### The expert panel

The expert panel was comprised of those individuals who were central to developing and managing the largest European trauma registries; the panel included clinicians, database managers and epidemiology experts.

### Data variable definition

A data variable should be unambiguously defined (with no misinterpretations) and reasonably simple to register. To meet this requirement, a data variable dictionary should contain information on 'data point number,' 'data point name,' 'descriptive field name,' 'type of data,' 'data point category/value,' 'definition of data point,' 'source of data information' and 'coding guidance.' We based recommended guidelines for data variable definitions on existing trauma registry databases, the Utstein Template for Uniform Reporting of Data following Major Trauma [1], the US National Trauma Data Standard (NTDS) [36] and the Injury Surveillance Guidelines from the World Health Organization (WHO) [37].

### Core data variables

A registry should differentiate between data variables that absolutely need to be collected (core data) and the type of additional data that may be desirable (optional data) [1,37]. The current revision focuses on core data that are considered to be essential for documentation and reporting. We divided the core data into three groups ('Predictive Model,' 'System Characteristic Descriptors' and 'Process Mapping Variables') based on the role of the data variable in a registry.

### Predictive model

The predictive model is composed of patient and injury severity variables that are considered to be important for outcome prediction. Predictive models are not determinative; rather, they provide the probability of an outcome for a given patient [38]. Complex models, such as Abbreviated Injury Scale (AIS) [39] derivatives and the RTS, are often used to create such predictive models [38]. Experience from the German and UK trauma registries suggests that there may be better data variables to include in a predictive model than those traditionally used in the TRISS methodology [24,40-42].

### System characteristic descriptors

Data variables in the System Characteristic Descriptor group describe trauma systems. Within Europe, there are large differences in philosophies and structures of trauma care systems, and these data variables should indicate key differences between systems and permit comparisons of the effect of system structure on outcomes.

### Process mapping variables

Process mapping variables are intended to describe trauma care at an individual trauma centre (e.g., what happens to a patient after a major trauma); these are used for documentation of the patient journey, care process and care activities.

### Specific premises

At present, many trauma registries have difficulty in obtaining data for patients from all involved hospitals when patients are transferred between them; therefore, the expert panel based their consensus on the premise that the core dataset was intended to cover the main hospital where a patient is treated. However, the expert group recommended that all trauma registries develop methods to track patients through the trauma system and that both the primary (local) trauma hospital and the referral trauma hospital record the same set of core data variables. The introduction of a core outcome data variable will secure that the overall effect of the entire trauma system can be measured, even if part of the patient's treatment course is not recorded in detail.

### The nominal group technique

The modified NGT process consisted of four steps. First, each expert was supplied with necessary background documents (Table 1), and asked to return (by e-mail) proposals for inclusion and exclusion criteria, as well as a maximum of 30 core data variables in a prioritised order. This first proposal was summarised and structured by the coordinators (KGR, HML), and the collated results were redistributed in the second step for comments and re-prioritisation. The third step consisted of two consensus meetings in which members of the expert panel discussed their views in a structured way and then made conclusions. In the fourth step, the panellists were able to comment on the conclusions by e-mail. To complete the process, a letter of consent was signed by all experts.

**Table 1:** Attachments sent to the expert panel prior to the Utstein 2007 meeting.

**No.**	**Document name**
1	Dick et al. Recommendations for uniform reporting of data following major trauma – the Utstein style [1].
2	Conclusions from the Utstein symposium on 'Improving Trauma Systems and the Role of Trauma Registries'.
3	Inclusion and exclusion criteria and data points from the European Trauma Audit & Research Network.
4	The Swedish Trauma Registry Standard (KVITTRA), Data Dictionary.
5	The Norwegian National Trauma Registry, Data Dictionary.
6	American College of Surgeons, National Trauma Data Bank; National Trauma Data Standard, Data Dictionary v. 1.2 [36].
7	ICD-10, Chapter XX. External causes of morbidity and mortality [61].

## Results

The expert panel concluded that a New Injury Severity Score [43] (NISS) > 15 should be used as a single inclusion criterion (Table 2). Five exclusion criteria were listed (Table 2), and a total of 35 core data variables (23 in the predictive model group, eight system descriptors and four process mapping variables) were agreed upon (Tables 3, 4 and 5).

**Table 2:** Inclusion and exclusion criteria.

**Inclusion criteria**	NISS > 15.
**Exclusion criteria**	First hospital admission more than 24 hours after injury.
	Patients declared dead before hospital arrival, or with no signs of life on hospital arrival and no response to hospital resuscitation.
	Asphyxia.
	Drowning.
	Burn patients should be excluded if the burn represents the predominant injury, or if the patient is treated in a specialised burn unit.

NISS: New Injury Severity Score [43].

Signs of life: Pupillary response, spontaneous ventilation, presence of carotid pulse, measurable or palpable blood pressure, extremity movement, or cardiac electrical activity [51].

**Table 3:** Predictive model variables.

**Data variable no.**	**Data variable name**	**Type of data**	**Data variable categories or values**	**Definition of data variable**
1	Age	Continuous	Number	The patient's age at the time of injury.

2	Gender	Nominal	1 = Female 2 = Male 3 = Unknown	The patient's gender.

3	Dominating Type of Injury	Nominal	1 = Blunt 2 = Penetrating 3 = Unknown	Indication of the type of injury produced by the trauma.

4	Mechanism of Injury	Nominal	1 = Traffic: motor vehicle injury (car, pickup truck, van, heavy transport vehicle, bus) 2 = Traffic: motorcycle injury 3 = Traffic: bicycle injury 4 = Traffic: pedestrian 5 = Traffic: other (ship, airplane, railway train) 6 = Shot by handgun, shotgun, rifle, other firearm of any dimension 7 = Stabbed by knife, sword, dagger, other pointed or sharp object 8 = Struck or hit by blunt object (tree, tree branch, bar, stone, human body part, metal, other) 9 = Low energy fall (fall at the same level) 10 = High energy fall (fall from a higher level) 11 = Other 12 = Unknown	The mechanism (or external factor) that caused the injury event. The cut-off level for a fall should be defined as the person's height.

5	Intention of injury	Nominal	1 = Accident (unintentional) 2 = Self-inflicted (suspected suicide, incomplete suicide attempt, or injury attempt) 3 = Assault (suspected) 4 = Other 5 = Unknown	Information about the role of human intent in the occurrence of an injury, primarily determined by the incident and not by the resulting injury.

6	Pre-injury ASA-PS Classification System	Ordinal	1 = A normal healthy patient 2 = A patient with mild systemic disease 3 = A patient with severe systemic disease 4 = A patient with severe systemic disease that is a constant threat to life 5 = A moribund patient who is not expected to survive without the operation 6 = A declared brain-dead patient whose organs are being removed for donor purposes 7 = Unknown	The pre-injury co-morbidity existing before the incident. Derangements resulting from the injury should not be considered.

7	Pre-hospital cardiac arrest	Nominal	1 = No 2 = Yes 3 = Unknown	Did the patient suffer an injury-related pre-hospital cardiac arrest?

8	Glasgow Coma Scale (GCS) upon arrival of EMS personnel at scene	Ordinal	Number	First recorded pre-interventional GCS upon arrival at scene of medical personnel trained to assess.

9	GCS motor component upon arrival of EMS personnel at scene	Ordinal	6 = Obeys commands/appropriate response to pain 5 = Localising pain 4 = Withdrawal from pain 3 = Flexion to pain (decorticate) 2 = Extension to pain (decerebrate) 1 = No motor response	First recorded pre-interventional GCS motor component upon arrival at scene of medical personnel trained to assess.

10	GCS upon arrival in ED/hospital	Ordinal	Number	First recorded GCS upon arrival in the ED/hospital.

11	GCS motor component upon arrival in ED/hospital	Ordinal	6 = Obeys commands/appropriate response to pain 5 = Localising pain 4 = Withdrawal from pain 3 = Flexion to pain (decorticate) 2 = Extension to pain (decerebrate) 1 = No motor response	Fist recorded GCS motor component upon arrival in the ED/hospital.

12a	Systolic Blood Pressure (SBP) upon arrival of EMS personnel at scene	Continuous	Number	First recorded SBP upon arrival at scene of medical personnel trained to assess.

12b	SBP – clinical category – upon arrival of EMS personnel at scene	Ordinal	RTS 4 = >89 ("good radial pulse") RTS 3 = 76–89 ("weak radial pulse") RTS 2 = 50–75 ("femoral pulse") RTS 1 = 1–49 ("only carotid pulse") RTS 0 = 0 ("no carotid pulse")	First recorded SBP upon arrival at scene of medical person trained to assess.

13a	SBP upon arrival in ED/hospital	Continuous	Number	First recorded SBP upon arrival in the ED/hospital.

13b	SBP – clinical category – upon arrival in ED/hospital	Ordinal	RTS 4 = >89 ("good radial pulse") RTS 3 = 76–89 ("weak radial pulse") RTS 2 = 50–75 ("femoral pulse") RTS 1 = 1–49 ("only carotid pulse") RTS 0 = 0 ("no carotid pulse")	First recorded SBP upon arrival in the ED/hospital.

14a	Respiratory Rate (RR) upon arrival of EMS personnel at scene	Continuous	Number	First recorded RR upon arrival at scene of medical personnel trained to assess.

14b	RR – clinical category – upon arrival of EMS personnel at scene	Ordinal	RTS 4 = 10–29 ("normal") RTS 3 = >29 ("fast") RTS 2 = 6–9 ("slow") RTS 1 = 1–5 ("gasp") RTS 0 = 0 ("no respiration")	First recorded RR upon arrival at scene of medical personnel trained to assess.

15a	RR upon arrival in ED/hospital	Continuous	Number	First recorded RR upon arrival in the ED/hospital.

15b	RR – clinical category – upon arrival in ED/hospital	Ordinal	RTS 4 = 10–29 ("normal") RTS 3 = >29 ("fast") RTS 2 = 6–9 ("slow") RTS 1 = 1–5 ("gasp") RTS 0 = 0 ("no respiration")	First recorded RR on arrival in the ED/hospital.

16	Arterial Base Excess	Continuous	Number	First measured arterial base excess after arrival in the hospital.

17	Coagulation: INR	Continuous	Number	Use the first measured INR within the first hour after hospital arrival.

18	Number of days on ventilator	Continuous	Number	The total number of patient days spent on a mechanical ventilator (including all episodes). Record in full day increments with any partial day listed as a full day.

19	Length of stay in main hospital treating the patient	Continuous	Number	Calculate 'Date of discharge' minus 'Date of admission' from the reporting hospital.

20	Discharge destination	Nominal	1 = Home 2 = Rehabilitation 3 = Morgue 4 = Another CCU (higher treatment level) 5 = Another intermediate or low care somatic hospital ward 6 = Other 7 = Unknown	The patient's destination after end of acute care in the main hospital treating the patient. CCU = critical care unit.

21	Glasgow Outcome Scale – at discharge from main hospital	Ordinal	5 = Good Recovery 4 = Moderate Disability (Disabled but independent) 3 = Severe Disability (Conscious but disabled; depends upon others) 2 = Persistent vegetative state (unresponsive) 1 = Death 0 = Unknown	Glasgow Outcome Scale score at discharge from main hospital.

22	Survival status	Nominal	1 = Dead 2 = Alive 3 = Unknown	Alive or dead 30 days after injury.

23	Abbreviated Injury Scale (AIS)	Ordinal	Number	The AIS severity codes that reflect the patient's injuries. All injuries should be listed, even duplicated codes (e.g., bilateral femoral fractures, multiple spine fractures). The edition of the AIS coding dictionary should be indexed; AIS 2005 is recommended.

ASA-PS: American Society of Anesthesiologists Physical Status [65].

ED: Emergency Department.

EMS: Emergency Medical Services.

INR: International Normalized Ratio.

RTS: Revised Trauma Score [22].

**Table 4:** System characteristic descriptors.

**Data variable no.**	**Data variable name**	**Type of data**	**Data variable categories or values**	**Definition of data variable**
24	Time from alarm to hospital arrival	Continuous	HH:MM	The time between when the alarm call is answered (at the emergency call centre) and when the patient arrives at the reporting hospital.

25	Highest level of prehospital care provider	Ordinal	1 = Level I. No Field Care 2 = Level II. Basic Life Support 3 = Level III. Advanced Life Support, No Physician Present 4 = Level IV. Advanced Life Support On-Scene, Physician Field Care 5 = Other 6 = Unknown	The highest available level of competence of the pre-hospital care providers involved in the care of the injured patient.

26a	Pre-hospital intubation	Nominal	1 = No 2 = Yes 3 = Unknown	Was the patient intubated before arrival at the hospital?

26b	Pre-hospital intubation	Nominal	1 = A tube in the trachea (orotracheal, nasotracheal, or surgical airway) – drug assisted 2 = A supraglottic airway adjunct that prevents speech (such as esophago-tracheal combitube, the laryngeal tube, and various kinds of laryngeal masks)) – drug assisted 3 = A tube in the trachea (orotracheal, nasotracheal, or surgical airway) – not drug assisted 4 = A supraglottic airway adjunct that prevents speech (such as esophago-tracheal combitube, the laryngeal tube, and various kinds of laryngeal masks) – not drug assisted 5 = Other 6 = Unknown	Type of pre-hospital intubation. Drug assisted = anaesthesia, neuromuscular blocking drugs, and deep sedation.

27	Type of transportation	Nominal	1 = Ground ambulance 2 = Helicopter ambulance 3 = Fixed-wing ambulance 4 = Private/public vehicle 5 = Walk-in 6 = Police 7 = Other 8 = Unknown	Type of transportation delivering the patient to the hospital.

28	Type of first key emergency intervention	Nominal	1 = Damage control thoracotomy – (any emergency or urgent thoracotomy performed for bleeding or suspected bleeding into the chest, but excluding simple thoracic tube drainage) 2 = Damage control laparotomy – (any emergency or urgent laparotomy performed for bleeding or suspected bleeding into the abdomen, including bleeding from the aorta) 3 = Extraperitoneal pelvic packing 4 = Limb revascularisation (Arterial injury necessitating vascular surgery or interventional radiology, including all interventions for pulseless limb, decreased perfusion and intimal arterial injuries) 5 = Interventional radiology (Angiographic embolisation; Stent; Stent-graft placement – excluding limb revascularisations which are classified as 4) 6 = Craniotomy 7 = Intracranial pressure (ICP) device insertion (excluding cases were the ICP device was inserted as part of a craniotomy which are classified as 6)	The first key emergency intervention performed for the treatment and stabilisation of the patient's injuries.

29	Activation of the trauma team	Nominal	1 = No 2 = Yes 3 = Unknown	Was the patient met by an activation of the trauma team prior to or upon arrival at the hospital?

30	Inter-hospital transfer	Nominal	1 = No 2 = Yes – Transferred IN to the reporting hospital 3 = Yes – Transfer OUT of the reporting hospital 4 = Yes – Transferred both IN and OUT of the reporting hospital 5 = Unknown	Was the patient transferred from/to another hospital for acute treatment?

31	Highest level of in-hospital care	Ordinal	1 = Emergency Department 2 = General Ward 3 = Operation Theatre 4 = High Dependency Unit 5 = Critical Care Unit (definition based on nurse to patient ratio) 6 = Unknown	The highest level of care in the main hospital.

**Table 5:** Process mapping variables.

**Data variable no.**	**Data variable name**	**Type of data**	**Data variable categories or values**	**Definition of data variable**
32	Time from alarm to arrival at scene	Continuous	HH:MM	The time from when the emergency call is answered (at the emergency call centre) until the first medical provider (at least the equivalent of EMT's) arrives at the patient.

33	Time until normal arterial base excess	Continuous	HH:MM	The time from first measured arterial base excess at hospital admission until first measured arterial base excess within normal range. Reference range for base excess: ± 3 mmol/l.

34	Time to first CT scan	Continuous	HH:MM	The time from hospital admission until the time marked on the first CT scan image.

35	Time until first key emergency interventions	Continuous	HH:MM	The time from hospital admission until the FIRST emergency intervention. Record the time from hospital admission until the time of FIRST knife to skin is performed. Consider only the emergency interventions listed in data variable number 28.

CT: Computed Tomography.

EMT: Emergency Medical Technician.

## Discussion on inclusion/exclusion criteria and core data variables

### Inclusion criteria

NISS is a modification of the Injury Severity Score (ISS) method [43]. ISS is calculated by summing the squares of the highest AIS severity codes in each of the three most severely injured ISS body regions [44]. Hence, ISS will ignore all but the most severe injury in a body region, and often fails to consider worse injuries in other regions of the body [43]. In contrast, NISS is defined as the sum of the square of the three most severe AIS injuries regardless of body region [43]. Several authors have argued for replacing ISS with NISS [43,45-49]. Osler et al. considered NISS to be easier to calculate and more predictive of survival than the ISS method [43], and a recent study by Lavoie et al. confirmed their findings [46]. NISS will be equal to or greater than ISS for any given patient, and it appears to be a more accurate method for rating severely injured patients [49,50]; specifically, this is true for patients with multiple head injuries [46]. The increased number of included patients by choosing NISS > 15 instead of ISS > 15 should be seen as an increase in 'sensitivity' without a loss of 'specificity' of an ideal definition of major trauma. An effort should be made to secure that all patients with a NISS > 15 are included, regardless of whether or not the trauma team was activated prior to or upon the patient's arrival at the hospital, and whether or not the patient was admitted to an intensive care unit.

### Exclusion criteria

Using NISS > 15 as a single inclusion criterion will include some patients that are at high risk of confounding data analysis. To remove such patients from the analysis, a set of exclusion criteria was defined. The expert panel recommended excluding first hospital admissions more than 24 hours after the injury (e.g., prolonged search and rescue missions), patients declared dead before hospital arrival, or those with no signs of life (pupillary response, spontaneous ventilation, presence of carotid pulse, measurable or palpable blood pressure, extremity movement, or cardiac electrical activity) [51] upon hospital arrival and those having no response to hospital resuscitation. In addition, it was recommended that asphyxias, drowning and burns should be excluded (Table 2).

Pre-hospital deaths should be excluded for practical reasons, since in some countries patients declared dead in the pre-hospital setting are transported directly to the morgue; whereas in other countries, they are admitted to hospital. All patients who arrive in the ED with spontaneous circulation should be included, even if they have had a period of cardiac arrest before being admitted or if they die in the ED.

Asphyxia, drowning and burns are sufficiently different from blunt and penetrating injuries to require other datasets, and need to be considered separately. The UK National Burn Injury Database [52] is currently in use specifically for this purpose. Although the AIS 2005 edition has codes for asphyxia and drowning, such injuries were not included in earlier AIS editions, making comparisons across versions more difficult. In some (but not all) countries, major burn patients are sent to dedicated burn unit hospitals, thereby confounding comparisons. Burn patients should be excluded if the burn represents the predominant injury, or if a patient is treated in a specialised burn unit. In such patients, outcome is determined by factors other than those suggested in this paper. Including burn patients will not represent a sufficient number of patients to report on; hence, burn-related injuries will add little power to the predictive model.

### Predictive model variables

Age is an independent predictor of survival after trauma [53,54]. While the original TRISS model operates with only two age categories, current predictive models utilise different age groups, and we therefore recommend reporting the patient's nominal age (continuous) at the time of injury, in years without decimals, and always rounding down. Patients under one year of age should be reported with one decimal number (e.g., six months is 0.5).

Gender is recommended as a core data, since some studies have reported no association between gender and mortality after traumatic injury [55]; whereas others have found age-specific associations between male gender and outcome [56-58].

An evaluation of type of injury (blunt versus penetrating trauma) is useful for determining which patients are candidates for surgical haemostasis [59], and is essential in the TRISS model [8]. The previous Utstein document recommended that for cases involving both blunt and penetrating injuries, the predominant type of injury should be recorded [1]. The expert panel defined the dominating injury as the one with the highest AIS score. In the rare event of a patient having both blunt and penetrating traumas with the same AIS severity score, penetrating trauma is defined as the predominant injury.

The significance of the mechanism of injury (MOI) in prediction of trauma and outcome is, to a large extent, undetermined [60]. The MOI should be of value for epidemiology or subgroup analysis, and should be described in categories with reasonable prevalence rates. The International Classification of Diseases, 10th revision (ICD-10) [61], chapter XX, External causes of morbidity and mortality (V01-Y98), was initially examined for the purpose of the template; however, it was found to be too detailed, with too many injury codes. Therefore, the expert panel developed a reduced set of categories, which should make data collection easier. The set still enables the analysis of important subgroups, and since it is compatible with the ICD-10 codes, it will allow future category expansion if required.

In the ICD, most injuries can be grouped into two dimensions: intent and mechanism [62]. 'Intention of injury' provides information about the role of the human intent of an injury. The included list of categories is based on the ICD-10 codes, and is selected by the expert panel since it covers most injury intentions.

The presence of significant co-morbidity represents an independent predictor of mortality after trauma [1,53,63,64], and the expert panel recommends employing the American Society of Anaesthesiologists Physical Status (ASA-PS) classification system [65] for classifying the pre-injury co-morbidity status concretised by selected examples from the Norwegian Society of Anaesthesiology [66] (Table 6). The ASA-PS classification system is easy to use and employed daily by anaesthesiologists world-wide. The pre-injury ASA-PS classification system should be used solely to categorise co-morbidity that exists before injury [6]. Skaga et al. found this classification system to be a significant predictor of mortality after trauma, also when adjusting for the variables in the traditional TRISS model [6]. Physiologic derangement resulting from the present injury is not reflected in the pre-injury score, only co-morbidity existing before the incident [6]. The pre-injury ASA-PS is not identical to the preoperative or the post-injury ASA score from anaesthesia records.

**Table 6:** American Society of Anesthesiology Physical Status (ASA-PS) Classification System.

**ASA-PS 1**	**A normal healthy patient.**
Guidelines:	No organic, physiologic, biochemical or psychiatric disturbance. Any disorder is localized, without systemic effects. Smoking <5 cigarettes/day.
Example:	Healthy non-smoker, admitted for varicose vein operation.
**ASA-PS 2**	**A patient with mild systemic disease.**
Guidelines:	Present pathology might imply specific measures or anaesthesia related precautions. The disturbance(s) might be caused by the condition to be surgically treated or by another pathologic process. Smoking >5 cigarettes/day.
Examples:	Mild organic heart disease. Uncomplicated diabetes mellitus (type 1 or 2). Benign hypertension without complications. Healthy patient with trismus.

**ASA-PS 3**	**A patient with severe systemic disease.**
Examples:	Diabetes mellitus with organ complications. Disabling heart disease. Moderate to severe respiratory disease. Angina pectoris. Myocardial infarction >6 months ago.

**ASA-PS 4**	**A patient with severe systemic disease that is a constant threat to life.**
Guidelines:	The disease is not necessarily related to the condition to be surgically treated, neither is it necessarily improved by the surgical intervention per se.
Examples:	Malignant hypertension. Myocardial infarction <6 months ago. Severe liver, kidney, respiratory, or endocrine dysfunction. Manifest cardiac failure. Unstable angina pectoris. Subarachnoid haemorrhage – patient awake or somnolent.

**ASA-PS 5**	**A moribund patient who is not expected to survive without the operation.**
Examples:	Patient in circulatory shock because of ruptured aortic aneurysm. Deeply comatose patient with intracranial haemorrhage.

**ASA-PS 6**	**A declared brain-dead patient whose organs are being removed for donor purposes.**

The six ASA-PS headings are from the American Society of Anaesthesiologists [65]. The guidelines and examples were translated from the Norwegian edition [66] by Skaga et al. [6].

For the Utstein Template, the ASA-PS classification system should solely be used to categorise pre-injury comorbidity. Derangements resulting from the injury should not be considered.

Pre-hospital cardiac arrest after trauma has dismal outcomes [67-70]; it was therefore chosen as a core data variable.

The panel recommends using raw values (continuous data) of the GCS, SBP, RR, where they are obtainable, and coded values (clinical categories) according to the RTS [22] (Table 7) in those cases missing raw values. In addition, the expert panel recommends recording the first pre-hospital, pre-interventional GCS, SBP and RR collected by a trained person, as well as the first GCS, SBP and RR upon arrival at the hospital.

**Table 7:** Revised Trauma Score (RTS) categories with clinical notes.

**RTS coded values**	**Respiratory Rate**	**Systolic Blood Pressure**	**Glasgow Coma Scale score**
4	10–29 ("normal")	>89 ("good radial pulse")	13–15

3	>29 ("fast")	76–89 ("weak radial pulse")	9–12

2	6–9 ("slow")	50–75 ("femoral pulse")	6–8

1	1–5 ("gasp")	1–49 ("only carotid pulse")	4–5

0	0 ("no respiration")	0 ("no carotid pulse")	3

This table is based on (but not identical to) the RTS table in reference [22]. The parentheses represent clinical notes that were added by the expert panel.

For patients intubated before arrival at the hospital, the ED GCS is not attainable, and this could potentially result in large amounts of missing data [18]. For this reasons, the pre-hospital, pre-interventional GCS should be recorded, thus permitting its inclusion in future prediction models. Some studies have shown that the pre-hospital GCS score correlate well with the arrival GCS score [71,72], and that the pre-hospital GCS score is a strong predictor of outcome [73]. A change in the GCS from the field to hospital arrival is highly predictive of outcome [72,74]. In the RTS, GCS carries the greatest weight [22], and the motor component of GCS appears to contain most of the predictive power of the total GCS score [75]. This component needs further assessment, something that may be facilitated by reporting both the pre-hospital pre-interventional GCS motor component as well as the first recorded GCS motor component upon arrival in the ED/hospital.

Chan et al. found that even in the case of normal SBP upon arrival in the ED, out-of-hospital hypotension was a clinical predictor of severe injury [76]; it was also a strong predictor of the need for emergency surgery, according to Lipsky et al. [77]. Others have documented an increased risk of intra-abdominal injury in blunt trauma patients with hypotension in the hospital [78,79]. Franklin et al. reported 16% mortality in patients with a period of pre-hospital hypotension but with stable vital signs on ED presentation, vs. 27% mortality for patients with normal pre-hospital SBP who developed hypotension in the ED [80]. Changes in the SBP values, from the pre-hospital pre-interventional value to the SBP value on hospital arrival, could give valuable information about physiologic derangements.

Missing RR values for patients arriving in the hospital (patients intubated before arrival) is the most common cause of a lack of RTS values [81]. Collecting the pre-hospital, pre-interventional RR as well as allowing the use of coded values instead of precise values may compensate for this [18]. In addition, continued documentation of RR for later determination of its predictive power is highly recommended. Creating alternative predictive models may also necessitate the continuous collection of RR values.

Admission arterial base excess (BE) is a predictor of mortality after trauma [40,82-84]; it is also considered an indicator of haemodynamic instability, high transfusion requirement, and an indicator of metabolic and coagulatory decompensation in trauma patients [40,85]. Other results indicate that arterial BE is a predictor of intra-abdominal injury [78,79], and the European guidelines for management of bleeding following major trauma have recommended arterial base deficit as a sensitive test to estimate and monitor the extent of bleeding and shock [59]. Kroezen et al. showed that replacing RTS by base deficit as a measure of physiological disturbance could predict mortality as well as RTS in the TRISS model [86]. The expert panel recommends recording the first arterial BE after arrival in the hospital.

There is a high frequency of established coagulopathy in multiple-injured patients upon their arrival in the ED [87,88]. Coagulation abnormalities presenting early after trauma have been found to be an independent predictor of mortality [87-90], and the prognostic value of measuring coagulation parameters upon ED admission has been documented [89]. The first measured International Normalized Ratio (INR), obtained within the first hour after hospital arrival, was chosen as a core data for measuring coagulation abnormalities.

The term 'intensive care unit' (ICU) is variously defined across the world, and in some referral hospitals, many patients are intubated upon arrival or transferred back to a local hospital while still intubated and ventilated. This makes the calculation of length of stay (LOS) in the ICU difficult. The revised template recommends recording the total number of days spent on a mechanical ventilator, rather than ICU LOS, as a measure of resource consumption. Furthermore, these data permit the use of the concept of ventilator-free days to quantify morbidity after trauma.

As outcome measures, the expert panel suggest hospital LOS, discharge destination, Glasgow Outcome Scale (GOS) [91] score at hospital discharge and 30-day mortality. The GOS contains information about morbidity, and this data collection represents an increased focus on information regarding morbidity. In the original US MTOS, "end of acute care" was used as an outcome measure [9]; however, 30-day mortality is considered to be a more fixed endpoint than "end of acute care/hospital discharge" or "end of somatic care," which will vary depending on the transfer and rehabilitation policies of an individual system [92]. Using 30-day mortality is consistent with the previous Utstein recommendations [1]. Death occurring later than 30 days after injury is more likely to be caused by other conditions, such as pre-existing disease [81]. An analysis of 69,650 patient admissions from the TARN showed that 4.8% of the patients died within 93 days of admission [41]. Of these, only 9% died later than 30 days after admission; these were mainly patients with a low ISS (< 9) and aged > 65 years. In a Scandinavian trauma registry, 4.6% of the deaths occurred later than 30 days after injury [92], whilst data from the German trauma registry (cases with NISS ≥ 16) indicate that 4.9% of the patients that died did so later than 30 days after injury [personal communication with the DGU-TR]. Survival status of all patients at a single given endpoint is needed, and 30-day mortality was chosen to represent this information.

For injury severity description, the expert panel recommends recording all AIS codes. It has previously been shown that a comparison of survival for trauma registries that use different AIS editions is potentially possible [93]. A difference in registrars and different levels of AIS training probably represents a greater problem than problems with using different AIS versions. However, registries are recommended to use the same AIS edition as a uniform way of coding; hence, the newest available version should be used at all times. At present, this is the AIS 2005 edition.

### System characteristics descriptors

The expert panel considered the time between when the alarm call is answered (at the emergency dispatch centre) and when the patient arrives at the first hospital to be an important system characteristic descriptor (this variable can also be useful for mapping the entire process of pre-hospital rescue) and recommend that this interval be reported.

Recording the highest level of competence of the pre-hospital care providers was regarded by the expert panel as an important measure for describing the pre-hospital system, and for cross-border comparisons of pre-hospital trauma care and outcome. The highest available level of the providers may vary somewhat in Europe, but the revised template's categorisation of level of provider is based on the levels proposed by McSwain [94], since these levels will encompass most pre-hospital systems.

Pre-hospital intubation is an important parameter that represents pre-hospital advanced life support (ALS) [95]. There is an ongoing debate on the use and role of ALS [96-98] measures in the out-patient management of trauma victims, and such information should be made available for possible inclusion in future survival prediction models. The original US TRISS model was derived from a dataset that excluded trauma patients who were intubated out-of-hospital [9]. However, these patients constitute a significant proportion of European trauma victims; hence, the expert panel recommends including them. In a study by Arbabi et al., early field intubation was associated with a decreased risk of fatal outcome, as compared to ED intubation [71]. In their study, intubation status was also an independent predictor of fatal outcome, after adjusting for ISS, SBP, mechanism, age and ED-GCS [71].

The type of transportation delivering the patient to the hospital is an important descriptor of the pre-hospital trauma care system, but the use of a helicopter vs. ground ambulance remains controversial [99,100], and assessment is confounded by differences in various EMS and HEMS systems. Since transportation type has been so widely debated, this is recommended as core data to be collected. The data variable does not cover what is a commonly used transport combination in some parts of Europe; ground ambulance to the local hospital and fixed-wing or rotary-wing transfer to the regional trauma centre. The data variable was developed to cover the type of transportation delivering the patient to the reporting hospital.

The expert panel recommends registering the initial key emergency intervention (EI) conducted during the hospital stay (ED, OR, critical care unit). These interventions (Table 8) represent essential emergency procedures used for the treatment and stabilisation of patients with severe injuries. Some registries will probably collect finer resolution data on interventions, but most patients will fit into one of these categories. It is recommended that the EI found in the present categories be recorded, even if there is no proof in a patient's notes that the cause of intervention was bleeding. The term "damage control", as used in some categories, implies that only the urgent (rather than later planned procedures) should be recorded. All limb revascularisations should be categorised in the same group (whether surgical or radiological) since it is the fact that the patient needed revascularisation (rather than the exact method used) that is important. If radiological revascularisations are put together with all the rest of the interventional radiology, the frequency of revascularisations undertaken in a particular system will not be easily measurable. Information about the surgical revascularisations will not permit comparison of the need for revascularisation across systems. The need for revascularisation is a more interesting comparison than comparing the way in which revascularisations were achieved. Extraperitoneal pelvic packing for control of massive traumatic pelvic haemorrhage has been described by several authors [101-103], and is a separate EI subcategory. Rapid intervention is essential for patients with intracerebral haematomas that require evacuation; for patients requiring only intracranial pressure (ICP) monitoring as part of the overall intensive care management of diffuse brain injuries [104], however, this intervention would not have the same level of urgency. Nevertheless, insertion of an ICP monitoring device will provide information about neurosurgical alertness in a trauma system, and is therefore included. The suggested list of EI was developed to cover the broad majority of emergency interventions (but perhaps not all of them). For the purpose of comparative evaluation of the acute treatment process, some less common types of intervention may not be appropriate.

**Table 8:** Key emergency interventions.


**No.**	**Emergency interventions**

1	Damage control thoracotomy – (any emergency or urgent thoracotomy performed for bleeding or suspected bleeding into the chest, but excluding simple thoracic tube drainage)

2	Damage control laparotomy – (any emergency or urgent laparotomy performed for bleeding or suspected bleeding into the abdomen, including bleeding from the aorta)

3	Extraperitoneal pelvic packing

4	Limb revascularisation (Arterial injury necessitating vascular surgery or interventional radiology, including all interventions for pulseless limb, decreased perfusion and intimal arterial injuries)

5	Interventional radiology (Angiographic embolisation; Stent; Stent-graft placement – excluding limb revascularisations which are classified as No. 4)

6	Craniotomy

7	Intracranial pressure (ICP) device insertion (excluding cases were the ICP device was inserted as part of a craniotomy, which are classified as No. 6)

A formal trauma team is an essential part of an organised trauma system [104]. Activation of the trauma team is an organised initial response to a trauma [104,105] with the primary goal of securing fast and efficient treatment of severely injured patients [106-108]. Information on whether the trauma patient was met by an activation of the trauma team was recommended as vital for describing a trauma system.

The revised template recommends recording the highest level of in-hospital care for the trauma patient in the main trauma hospital (Table 4).

### Process mapping variables

The "Chain of Survival" concept emphasises that all time-sensitive interventions must be optimised to maximise the chance of patient survival [109]. The expert panel recommends recording the time between when the emergency call is answered (at the emergency call centre) and when the first medical provider arrives at the patient. This core data represent parts of the first link in that chain, and is an important measure of the quality of the pre-hospital EMS system.

As an overall marker of the efficiency of patient treatment (including resuscitation, diagnostics and surgery), the expert group suggested to consider the time required to achieve normal arterial BE. A good evidence basis does not exist for this recommendation, however, if BE worsens after arrival, prognosis worsens as well [110]. The BE should be measured regularly after hospital arrival, and the template advises to document the initial measurement of BE immediately after hospital arrival, and in cases of abnormal values, to document the time in hours until normalisation.

The efficiency of the initial in-hospital management is assessed by the time from hospital admission until the time marked on the first CT scan images. This data represents the time required to perform key in-hospital diagnostic tests.

The time to the first key EI should be recorded; this measure represents how quickly an urgent intervention fundamental to the treatment and stabilisation of a patient's specific injuries [105] is performed. This core data measures the efficiency of the trauma system in the initial phase. The time elapsed between injury and EI should be minimised.

## General discussion

The present paper represents a further development of the previous Utstein Template for Uniform Reporting of Data following Major Trauma [1], and reflects a need for the creation of new prediction models that are more suitable for the type of trauma seen in Europe. Currently, both the German and UK trauma registries have developed their own predictive models, and no longer use the original TRISS system. However, uniform and standardised inclusion and exclusion criteria and a core list of data variables with precise definitions are mandatory before comparisons between trauma registries can be made. The expert panel reached a consensus on such a list of core data. This dataset does not preclude the possibility that in local trauma registries more data can legitimately be considered core information for specific purposes. The data agreed upon in the present process represent data for admissions to the first hospital within 24 hours after injury. We are aware that some countries have made further progress in developing national trauma systems and national trauma registries; whereas others only register information at local trauma hospitals. Currently, the core dataset will be difficult to use to assess the entire trauma system of some regions or countries, making direct system comparisons difficult. At this point, the expert panel recommends focusing on the hospitals of definitive treatment, and registering patient transfers (as a separate data variable) should allow for this when data are analysed. The inclusion of 30-day mortality in predictive models allows for the assessment of more of the total system performance. However, excluding pre-hospital deaths and the details of management along the chain that precede the main treatment hospital represents a major limitation in total system assessment. The core dataset should undergo further development to allow for routine tracking of the patient through a trauma system consisting of more than one hospital. We anticipate that future development of the Utstein Template will focus more on the trauma system as European trauma registries develop, making it possible to follow transferred patients. In future trauma systems, the same core data variables should be recorded in primary, secondary and tertiary trauma centres.

The template classifies fall-related injuries as low or high (category options of the MOI data variable in Table 4) with a person's height as the cut-off value. Various definitions of falls are used internationally [61,111-115]; they range from different categories of height, different cut-off levels for height, to differing units of height measurement (feet vs. meters). This is an area in which there is a lack of uniformity; therefore, for future development, we suggest that individual registries should also record the actual estimated height of a fall in meters (as a continuous variable), so that an analysis can be performed.

We are aware that the best way of assessing the GOS as a measure of disability (morbidity), is after at least six months, but since this was not considered feasible, registration at discharge from the hospital was chosen as the endpoint. The assessment of GOS as an outcome measure at discharge from a trauma centre in trauma systems based on early discharge to specialised rehabilitation services represent a limitation, and the GOS endpoint should therefore be interpreted with caution. Nevertheless, assessment of this endpoint data can be used as a rough estimate of the amount of care needed for a patient beyond the acute hospital stay. Although GOS was developed for patients with head injuries, it represents a rough disability outcome score, and as such it should be possible to use for assessing all trauma patients.

The revised Utstein Template considers time to normalised arterial base excess as a measurement of importance for evaluating the total quality of trauma treatment. This data is not a precisely timed measurement and will probably vary from hospital to hospital. However, collecting it is considered valuable and might be important for future comparisons. The inclusion of this data also has some educational reasons; the trauma centres should be encouraged to include this value regularly in the trauma patients' charts.

The expert panel recommends reporting the first EI, and the time to first EI, disregarding the fact that some patients may have received several of the listed interventions. The first intervention is, by clinical judgement, the most important one for comparison. This does not preclude trauma registries from documenting each of these interventions separately, or even each operation that is performed. However, the type of first EI and the time elapsed prior to its application is of significant importance; for this reason, this measure was chosen as part of the core dataset. For the purpose of comparison, focusing on the first EI performed in the current system will be adequate. However, if comparisons based on all EI are desired, the present recommendation will not be adequate. Comparison of systems based on all interventions can only be used if it is coupled with data on the time of each intervention. This will be difficult to use if the order in which the interventions occur is not known. If the first intervention is not among those listed under the EIs in the template, time to intervention is not important in our context (i.e., there will be enough time for thorough surgical preparation), and it should therefore not be part of the Utstein core dataset.

The panel acknowledges that no indicator has 100% validity and careful judgement is therefore needed. For example, in some selected cases earlier emergency surgery may imply both later CT imaging and better practice. Definition of data variables is a complex and ongoing process and in order to widen the implementation of the core dataset, facilitate participation in European trauma audit and comparisons and increase the quality of the next updates of the core dataset, we encourage all readers to ask for clarifications and point out potential improvements.

One concern with the Utstein Template is implementation, since none of the participants from the previous Utstein trauma process changed their registries to accommodate the recommendations of that meeting. The solution for the revised template was to develop a letter of consent that all members of the expert panel signed where they agreed on implementing the core data in the revised Utstein Template [see Additional file 1]. To further facilitate implementation, a complete user manual with core data variables and variable definitions will be available free of charge, at: , , , .

## Conclusion

This paper represents a major step in perfecting the Utstein Template for Uniform Reporting of Data following Major Trauma, making the core data variables more uniform and applicable. Collecting this core dataset should be a basic component of all future studies on trauma care, and a uniform dataset such as this, will facilitate accurate description of the patient population and allow comparisons of outcome from trauma systems. It is extremely important that the data variables are collected in a uniform manner. For this reason, each variable and response category has been specifically defined in a way that is designed to promote the collection and reporting of a comparable core dataset. A letter of consent has been signed by the expert panel, where the participants of this consensus process agreed to implement the inclusion and exclusion criteria and core data variables in their respective systems and registries.

## Competing interests

The authors declare that they have no competing interests.

## Authors' contributions

KGR and HML designed the study and organised the consensus meetings. TJC was the chairman of the consensus process, while LH and OR were co-chairmen. The core dataset was developed by the expert panel. KGR, TJC, RL, SDB, OR, LH, PAS and HML wrote the article. The rest of the panellists read the manuscript once, made comments to it and approved the manuscript. All authors read and approved the final version of the manuscript.

## Supplementary Material

Additional file 1 Letter of Consent. The letter provided was signed by the Utstein TCD expert panel. With this letter, the expert panel members confirm that they will implement the core data agreed upon. Click here for file
