# Supplementary material for: The Utstein template for uniform reporting of data following major trauma: A joint revision by SCANTEM, TARN, DGU-TR and RITG
Source: Scand J Trauma Resusc Emerg Med. 2008 Aug 28;16:7. doi: 10.1186/1757-7241-16-7 (PMC2568949; doi:10.1186/1757-7241-16-7)
Supplement: Additional file 1 — Letter of Consent. The letter provided was signed by the Utstein TCD expert panel. With this letter, the expert panel members confirm that they will implement the core data agreed upon. [file 1757-7241-16-7-S1.pdf]

Letter of Consent

From April to December 2007, selected representatives from different trauma organisations and networks in Europe participated in a modified nominal group technique process, which included two consensus-meetings at the Utstein Abbey in Norway. The mission was to agree on a European standard of inclusion and exclusion criteria and a minimal core dataset, for documenting and reporting data following major trauma. A consensus was reached, and with this letter, we, the participants of the Utstein consensus process hereby confirm that we will implement the inclusion/exclusion criteria and the core data points agreed upon.

Timothy J. Coats
Trauma Audit & Research Network

Olav Røise
Ullevål University Hospital

Lauri Handolin
Töölö Hospital Trauma Registry,
Helsinki University Hospital

Hans Morten Lossius
Stavanger University Hospital &
Norwegian Air Ambulance

Kjetil G. Ringdal
Norwegian Air Ambulance &
Ullevål University Hospital

Rolf Lefering
Trauma Registry of the German Society of Trauma Surgery

Stefano Di Bartolomeo
University of Udine,
Udine, Italy

Maaret Castrén
Södersjukhuset,
Karolinska Institutet

Torsten Eken
Aker University Hospital

Erika F. Christensen
Emergency Medical Services
Central Denmark Region

Per Örtenwall
KVITTRA

Nils Oddvar Skaga
Trauma registry
Ullevål University Hospital

Lena Klarin
KVITTRA

Ernestina Gomes
Hospital Geral de Santo António, Porto

Morten Schultz-Larsen
Trauma registry
Odense University Hospital

Maralyn Woodford
Trauma Audit & Research Network

Morten Hestnes
Trauma Registry
Ullevål University Hospital

Antoinette Edwards
Trauma Audit & Research Network

Torben Wisborg
Hammerfest Hospital

Ari Leppäniemi
Meilahti Hospital
Helsinki University Hospital
